# Identifying and Addressing Genetic Counseling Challenges among Indigenous People of Oaxaca—One Center’s Experience with Two Immigrant Farmworker Families in the Central Valley of California

**DOI:** 10.1007/s10897-018-0221-0

**Published:** 2018-02-03

**Authors:** Joseph J. Shen, Jason Carmichael, Leoncio Vásquez Santos

**Affiliations:** 1 Division of Genetics, Department of Pediatrics, University of California, San Francisco – Fresno, 290 N. Wayte Lane, Fresno, CA 93701 USA; 2 Medical Genetics and Metabolism, Valley Children’s Hospital, Madera, CA USA; 3 Binational Center for the Development of Oaxacan Indigenous Communities, Fresno, CA USA

**Keywords:** Genetic counseling, Underserved population, Immigrant population, Indigenous ancestry, Indigenous languages, Indigenous community, Oaxaca, Mexico, Mixtec people, Mixtecos, Traditional medicine beliefs, Multicultural genetic counseling

## Abstract

An important aspect of genetic counseling is the recognition of and adaptation to the socio-cultural uniqueness of the different populations that a genetics clinic serves. The Central Valley of California is home to a large population from Mexico, with a significant proportion of indigenous ancestry originating from the state of Oaxaca. We report on our experience with two families of this community—one extended family with an early lethal inborn error of metabolism and the other with a chronic disfiguring form of ichthyosis. We identified multiple important factors that needed to be considered, including the matching of language dialects, adaptation to different social interaction conventions, acknowledgement of traditional medicine beliefs, and effective transmission of genetic terms and concepts, all of which should be incorporated into the interactions with these families when aiming to provide comprehensive genetic counseling.

## Introduction

Among the Latin American population in the Central Valley of California is a significant proportion who originate from the Mexican state of Oaxaca, which has a large indigenous population. There are 16 recognized indigenous communities with numerous different languages (Caballero-Morales 2013). The Mixtec people, or Mixtecos, are more highly represented in the Central Valley because this area’s prominent agricultural industry matches the region from which they immigrated. Mixtec language can be divided into three groups: Mixteco Alto, Mixteco Bajo, and Mixteco De La Costa. However, these broad categories do not necessarily mean that two speakers identified as being part of the same group are matched well linguistically. There are regional dialects, best categorized and identified by their “distrito” (district), which is then subdivided into different “municipios” (municipalities), each with their specific colloquialisms and vernacular, which should also be matched to help optimize the verbal transmission of information.

There are multiple barriers in providing medical care to the Mixteco community that are similar to the challenges present in servicing the general Mexican immigrant population (Browner et al. 2003). Complicating factors include immigration status, socio-economic pressures, geographical distance, and educational level. Traditional medicine beliefs are also widely held throughout Mexico and are an integral part of their belief system. Elements of this practice include accessing “curanderos,” having additional explanations for medical problems (evil eye, curses, witchcraft, etc.) known and suffered by the communities, and subscribing to alternative therapies and cures (Barragan et al. 2011; Barron et al. 2004; Gonzalez-Vazquez et al. 2016). Recognition and acceptance of Mixteco individuals’ worldview optimizes the medical care provided by incorporating their psychosocial well-being into the treatment of their medical condition (Barragan et al. 2011; Barron et al. 2004; Gonzalez-Vazquez et al. 2016).

There are additional barriers due the fact that the Mixtecos are a minority community in Mexico. Similar to the situation with aboriginal and indigenous populations throughout the world, it is inaccurate to assume that they have assimilated into the dominant culture of their country. Although the natural tendency is to treat all immigrants who follow the same immigration routes and patterns as a homogeneous population, significant differences remain. Even if the indigenous subpopulation is recognized, generally there is a broader unfamiliarity with their customs and beliefs. Additionally, the challenges can be greater when discussing the basic tenets of genetics because the concepts of “genes,” inheritance patterns, and other similar topics cannot be understood given the educational background and the fact that equivalent terms in many languages simply do not exist (Butler et al. 2011; Dukepoo 1998; Kelly 2009; Kowal et al. 2015; Mittman et al. 1998; Penn et al. 2010; Port et al. 2008; Raz and Atar 2003; Solomon et al. 2012). The widely varying dialects and severe lack of medically qualified interpreters present further communication barriers. This makes the utilization of a cultural broker (someone familiar with the culture and language of both the clinical team as well as that of the target population, who can help bridge the cultural and linguistic differences) vitally important because genetic counseling is predicated on effective communication and building rapport (Jezewski and Sotnik 2001; Kowal et al. 2015; Mittman et al. 1998; Penn et al. 2010; Port et al. 2008). Recognition, acceptance, and incorporation of cultural differences into the genetic counseling session optimize the delivery of appropriate care (Catz et al. 2005; Chapple et al. 1995; Richards and Ponder 1996; Sue and Sue 2015; Wang and Marsh 1992; Wang 2001; Weil and Mittman 1993; Weil 2001).

This article describes the challenges encountered and the techniques utilized as we attempted to provide comprehensive genetic counseling and culturally sensitive care for two Mixteco families. One large family group consisted of two closely related sets of parents who in total had three children affected with an inborn error of metabolism that typically leads to death in infancy. The second family had two children who manifested a visually striking and readily apparent skin condition that was clinically diagnosed and molecularly confirmed after the birth of their second child in the USA. Our cultural broker was a Mixteco community advocate who also served as the interpreter [author L. Vasquez Santos, Executive Director of the Fresno-based Binational Center for the Development of the Indigenous Communities (CBDIO, its acronym in Spanish)].

## Clinical Information

### Family A

Patient A1 was born to a 22-year-old G1P0- > 1 mother after an uncomplicated pregnancy via C/S due to large for gestational age status. The parents did not report known consanguinity. California state-expanded newborn screening revealed a complex pattern, including a low methionine level of 5 μmol/L (cut-off is less than 8). Follow-up labs showed a methionine level of 3 μmol/L (normal > 10) and a total plasma homocysteine level of 246.6 μmol/L (normal < 10). He was started on betaine and cyanocobalamin for a possible cobalamin synthesis defect while the work-up continued. *MTHFR* sequencing (All Children’s Hospital, St. Petersburg, FL) showed homozygosity for the “A1298C” c.1298A > C (p.Glu429Ala) functional polymorphism and also homozygosity for a c.177G > T (p.Try59Cys) variant with conflicting in silico algorithm prediction results regarding its pathogenicity. Carrier testing was recommended but could not be completed. Based on this molecular information, in combination with the clinical presentation and biochemical abnormalities, the patient was diagnosed with severe MTHFR deficiency.

There were numerous challenges to providing comprehensive care that were identified early. It was readily apparent during the initial interactions that there was language difficulty, and the parents demonstrated very limited understanding of inborn errors of metabolism, metabolic management recommendations, and genetic diseases in general, yet did not ask any questions. They spoke an indigenous language, and a Mixteco Bajo interpreter from Silacayoapam, Oaxaca, certified for medical translation at our hospital, was used whenever possible, but sometimes, the discussions occurred in Spanish. The family inconsistently attended follow-up visits in the clinic, with some of the identifiable barriers being the distance they had to drive for clinic visits, and their intermittent stream of income due to seasonal work. A local public health nurse was utilized as much as possible to perform home health visits, ensure receipt of medications mailed to the home, and act as a local contact to provide assistance whenever needed.

Despite these interventions, there were still concerns as well as biochemical evidence that the family did not give medications to the patient as prescribed. During the frequent follow-up clinic visits, the parents were repeatedly counseled regarding natural history, anticipatory guidance, medications and dietary changes, and recurrence risks. They reported that their son’s early developmental milestones were normal up to at least 6 months of age. However, with serial clinical exams, he had clear neurological concerns including microcephaly, hypotonia, and perhaps subclinical abnormal movements that could be interpreted as seizures. He was admitted into the pediatric intensive care unit at 11.5 months of age because of pneumonia and respiratory failure. Overt seizures developed, and he continued to clinically decompensate. He became poorly responsive to environmental stimuli, whereupon discussions occurred with the family regarding his poor prognosis and to consider withdrawal of care. The family made requests centered around their traditional medicine belief system (see the “Results and Discussion” section), and he passed away at 12.5 months of age when medical care was withdrawn.

Patient A2 presented at age 2 months to an outside hospital with an acute life-threatening event consisting of choking episodes and respiratory difficulty. This is the second child for the parents (first child was healthy) born at term after an uncomplicated pregnancy via C/S because of large for gestational age status and failure to progress. The parents reported possibly noticing some staring episodes, but otherwise, they did not report any overt tonic-clonic movements or neurological concerns. Their son was transferred to the PICU at our hospital because of suspected aspiration pneumonia, possible seizures, brain MRI showing diffuse atrophy with delayed myelination and prominent extra-axial fluid spaces, and his worsening respiratory status. On obtaining the family medical history, the family divulged that patient A2 was a double first cousin of patient A1, who had passed away 8 months ago; patient A2’s father was the brother of patient A1’s father, and patient A2’s mother was the sister of patient A1’s mother. Review of his state newborn screening results showed a methionine level of 10 μmol/L, which was not flagged as it was above the cut-off of 8 μmol/L. The a priori chance that patient A2’s parents would have a child with MTHFR deficiency was 1 in 16, but with patient A2’s early neurological presentation and the borderline state newborn screening result, a total plasma homocysteine level was performed which was 148 μmol/L (normal < 10). Sequencing of *MTHFR* showed the same variants as noted for patient A1. His neurological and respiratory status deteriorated rapidly, and care was withdrawn just before 3 months of age.

Patient A3 was the younger sibling of patient A1, whose parents did not opt for prenatal genetic testing despite being informed about the recurrence risks and the prenatal testing options available. He was born after an uncomplicated pregnancy and delivery but flagged on state newborn screening with a low methionine level. Discussions were still ongoing for in-home hospice when he presented to the PICU at 1.5 months of age with respiratory failure. He was noted to be markedly hypotonic, and withdrawal of care occurred a few days later.

### Family B

Patient B was born after an uncomplicated pregnancy via emergency C/S because of placental abruption at 36 4/7 weeks. Abnormal skin findings were readily appreciated consisting of taut brown-colored cracked skin over her entire body. Family medical history was notable for remote consanguinity and an older sister (still living in Mexico) with similar skin findings at birth; she is now 11 years old, and a picture was viewed which confirmed the parents’ report that she has very taut skin and other dysmorphic features. A homozygous *TGM1* c.1437dupC frameshift variant consistent with lamellar ichythiosis was uncovered (Connective Tissue Gene Tests, Allentown, PA). Patient B was sent home with instructions on proper skin care and close follow-up with dermatology.

## Genetic Counseling Visits

### Family A

The numerous previous interactions with the two sets of parents for patients A1, A2, and A3, both in the outpatient clinic and while their children were in the PICU, included the hospital-based Mixteco Bajo interpreter whenever possible. A few months after patient A3 had passed away, a comprehensive genetic counseling approach was planned with L. Vasquez Santos, and then we met with both sets of parents together in an outpatient setting. L. Vasquez Santos functioned as the interpreter and cultural broker, and during this clinic visit, feedback was solicited regarding the genetics information and care that was provided.

### Family B

All three co-authors met with patient B’s parents for a genetic counseling session in the outpatient clinic after the molecular testing results had returned. A similar format was followed, with L. Vasquez Santos as the cultural broker and interpreter, and feedback also was requested during this visit.

## Results and Discussion

### Social Interactions and Building Rapport

#### Family A

The physician-family meetings and the genetic counseling sessions with the parents of patients A1, A2, and A3 did not proceed ideally for several reasons. One was that most of the discussions were conducted under stressful situations in an intensive care setting, and end-of-life decisions needed to be made in this environment within a compressed time frame. However, the initial contact with this family was in the outpatient clinic after patient A1 was identified on state-expanded newborn screening. Frequent visits occurred during which clinical and genetic counseling topics could be discussed gradually, such as the concept of disease, inborn errors of metabolism, medical management, and recurrence risks. During these interactions, it was identified early that another complicating factor was the parents’ indigenous background and that their primary language was not Spanish. Although a hospital-based Mixteco Bajo interpreter was utilized whenever possible, the interpreter voiced concerns, which were also apparent to the treating physicians who observed the conversations, that the flow of information was sub-optimal. It was suspected that the interpreter, who was from the municipality (and district) of Silacayoapam, Oaxaca, was an imperfect match with the parents’ dialect, as they came from a different district in Oaxaca approximately 53 km away (the municipality of Santiago Juxtlahuaca within the district of the same name). This region is where a co-author for this article (L. Vasquez Santos) is from, allowing him to act as an interpreter and cultural broker in preparation for, and to be a valuable part of, the final genetic counseling visit with the extended family.

Other factors contributing to less-than-ideal clinical genetics interactions and genetic counseling discussions were cultural differences in body language, eye contact, and expectations of bidirectional flow of information during these conversations. The parents rarely exhibited emotions, their eyes were mostly downcast, and they tended to provide short phrases or one-word answers, even for complicated and multifaceted medical questions and important care directives. The concept of “simpatia” with downcast eyes and minimal-to-no exhibition of emotion has been described previously (Barron et al. 2004) and is meant as a sign of respect towards the care providers. The parents also did not ask questions to clarify any concepts that may have been confusing to them; very likely, they did not consider questioning behavior to be appropriate because they felt it may be disrespectful to the medical authorities (Barragan et al. 2011; Barron et al. 2004). In contrast to the expectation in Western medicine for the family to be an active participant in the decision-making process, the culturally ingrained preference of the parents was for the providers to unilaterally make the medical decisions. This is how these interactions occurred during the metabolic clinic visits as an outpatient. However, in the intensive care unit setting and with “life or death” issues being discussed, there was confusion and doubt about whether the complex medical information was completely understood, and whether the parents were fully informed when making decisions and giving consents. A follow-up consultation in the genetics clinic was offered several months after patient A1 had passed away, but was declined at that time.

In contrast to the situation with the family of patient A1, the interactions with the parents of patient A2 were not preceded by outpatient clinic visits as he was identified as having severe MTHFR deficiency only after his clinical decompensation. Instead, in-depth discussions with his parents regarding his disease and the medical options available only occurred during the hospitalization prior to his death. Patient A3 was born a few months later and identified on the state-expanded newborn screen. There were only brief conversations by phone, and they centered around setting up hospice care before he presented to the PICU prior to his death. For both patients A2 and A3, the inadequate quality of the clinician-parent interactions was similar to that as described for patient A1, compounded by the fact that these conversations only occurred in the intensive care unit and were under more stressful circumstances, given the rapidly deteriorating clinical status and acuity of the underlying metabolic disease.

#### Family B

The initial interactions with patient B and her family occurred in the NICU. Likely due to acculturation differences for the father, his body language was different, as there was more eye contact and conversations had more bidirectional flow of information. On the other hand, the mother demonstrated more simpatia and deference to medical authorities, and she did not verbally engage. Overall, the parents were more accepting of this disease in their family, perhaps because they already have another affected child who was otherwise healthy aside from her physically apparent differences and need for appropriate skin care. The family came from the municipality of Coicoyan De Las Flores, which is somewhat geographically separated (~45 km) from Santiago Juxtlahuaca, but within the same district. L. Vasquez Santos was familiar with their dialect, and a test conversation by phone revealed a good level of mutual comprehension with the parents, informing us that he could interpret and participate fully in the genetic counseling visit and cultural discussions that subsequently occurred as an outpatient.

#### Final Genetic Counseling Visits

In contrast to the previous interactions with each of these families, it was apparent during the final genetics clinic outpatient visits with them that rapport was easily and quickly established with the co-author, L. Vasquez Santos. Verbal communication greatly increased, and the parents’ body language projected a more relaxed state. This increased comfort level, and cultural and linguistic familiarity, allowed us to effectively conduct a comprehensive genetic counseling session, but just as importantly facilitated a willingness for the families to ask questions and speak freely regarding their level of understanding as well as provide feedback.

The parents of patients A1, A2, and A3 stated that they believed what was being said to them by the clinicians, whether about the fact that their children had a severe disease despite the lack of overtly visible manifestations or that while in the PICU, their children were very sick and there was no cure available. They had no explanation for not fully following through with the metabolic management recommendations in the outpatient care of patient A1, nor was this issue pursued aggressively. However, it is suspected that the parents were utilizing and implementing traditional medical beliefs in conjunction with what was advised during the metabolic genetics visits (see the “Traditional Medicine Beliefs” section).

The parents of patients A1, A2, and A3, as well as the parents of patient B, freely admitted that the medical information and genetics concepts that were explained to them in the past were still very confusing. They agreed to hear the genetic counseling information that was prepared for the visit (see the “Discussion of Medical Genetics Concepts” section), perhaps out of politeness or some degree of curiosity, but they also indicated that a lack of complete understanding did not bother them. Overall, they did not express any regrets or anger towards the clinicians or the clinical course that led to their children’s demise (patients A1, A2, and A3) or physical disfigurement (patient B) (see the “Traditional Medicine Beliefs” section).

*Lessons learned*: (1) If an indigenous origin is noted for a family, one should identify the specific distrito (district) and municipio (municipality) to match with an interpreter speaking that dialect. It is less important that the interpreter may not be licensed specifically for medical terminology, especially because many genetic terms and concepts have no equivalent correlates in indigenous languages (Kowal et al. 2015; Solomon et al. 2012). Instead, the goal should be to inform the family adequately, and in a culturally sensitive manner, to empower them to understand the genetics care they are receiving, comprehend the ongoing clinical management, and participate more fully in the decision-making process. (2) Acculturation differences need to be taken into account, so that clinicians who are trained in the “Western style” of medicine can moderate their expectations of how the social and counseling interactions likely will proceed (Barragan et al. 2011; Kelly 2009). In some instances, similar to those previously reported (Browner et al. 2003; Kelly 2009; Mittman et al. 1998; Raz and Atar 2003; Smoot III et al. 1988), the medical and genetic staff may have to adapt to the deferential and unidirectional interactions that are a part of the family’s worldview, in which their desire may be for the providers to make the right decision on behalf of the family without any expectation of a non-directive decision-making process. Ideally, recruitment and utilization of a cultural broker (a relative with a greater degree of acculturation or a community leader or advocate) would incorporate both of these lessons and optimize the interactions with the family.

### Traditional Medicine Beliefs

#### Family A

The topic of traditional medicine was first broached during the outpatient clinical interactions with the parents of patient A1, but their answers were evasive and incomplete. It was in the pediatric intensive care unit and as part of end-of-life discussions that we were able to explore their ideas and wishes more fully. The parents actively solicited advice from an aunt who was a “curandero” (faith healer). She postulated that the origin of their child’s disease was from the mother being cursed due to an altercation that occurred either during her pregnancy or earlier in her life, and that there was “bad mixing of blood” from the mother and father. The aunt had a vision that various rituals were needed to be performed to help with his disease. This included prayer, an egg ceremony (passing an egg over the body to help draw out negative forces), and the burning of candles. Only this latter request could not be fulfilled because of the confines of the hospital room and presence of oxygen sources and sensitive equipment. The family also was granted a 14-day waiting period to allow for the possibility of a miracle to occur. Once it was accepted by the family that a cure was not forthcoming through either Western or traditional medicine, choices given to them included (1) performing a tracheostomy and discharge on a ventilator so that he could go home on hospice care, (2) being placed in foster care, or (3) withdrawing care. The parents did not want patient A1 to be apart from them and believed that the death of their child at home would draw in evil spirits, leading them to choose the last option.

The utilization of traditional medicine during the PICU stays of patients A2 and A3 was similar but of shorter duration compared to that of patient A1, as the closely related set of parents were very familiar with the events surrounding the death of patient A1. Their traditional medicine healers confirmed the Western medicine diagnosis of the children being afflicted by the same disease, with its associated poor prognosis. After the appropriate rituals were performed, there was more rapid acceptance of the next step of withdrawal of care.

During the final genetic counseling session, the two families of patients A1, A2, and A3 were more open about their thought processes and solicitation of additional medical advice during the clinical presentation of symptoms, disease diagnosis, and hospitalization ultimately resulting in each child’s death. Consistent with previous studies (Barragan et al. 2011; Barron et al. 2004; Gonzalez-Vazquez et al. 2016; Mittman et al. 1998; Penn et al. 2010; Shaw and Hurst 2008; Solomon et al. 2012), there was a duality to their worldview which took into account both Western medicine and traditional medicine beliefs. They stated that they believed what the treating physicians had said during the many discussions regarding the natural history of their child’s inborn error of metabolism, and while critically ill in the PICU. However, they did not accept the diagnosis of severe MTHFR deficiency as the only truth, with a recurring question being “Why is my child clinically getting worse, and why is there no cure [if the Western doctors are supposed to be knowledgeable and Western medicine is supposed to be correct]?”

Traditional medicine healers were accessed to help the families understand why their children developed this severe disease, interpret the ongoing and worsening illness, as well as to prescribe alternative cures. Besides soliciting advice from an aunt of the family who lived in the area, they utilized another independent curandero in their community, as well as a trusted source in Oaxaca with whom they spoke by phone. All of the healers were consistent with their answers in stating that likely the parents were cursed and that their children had advanced disease. When patient A2 started manifesting symptoms that seemed similar to that which led to the death of patient A1, it was suggested that there was the possibility of a curse not just impacting one of the parents but instead involving the extended family. The affected infants were microcephalic, so it was posited that their brains were not healthy enough for the growth and function of the entire body. Although the origin of the disease was felt to be certain and had identifiable etiologies, the only options presented by these healers to provide a cure were the prayers and different rituals mentioned previously.

An important aspect of their traditional medicine beliefs centered around the presence of witchcraft and an individual being cursed, and in particular, the “frightening” phenomenon (referred to as “ku’e niyuú in Mixteco Bajo”, and as “susto” or “asustado” in Spanish) (Penn et al. 2010; Poss and Jezewski 2002). The frightening was defined as an event leading to psycho-physiologic shock, which then stays with that person and subsequently can lead to negative health consequences later in life; there also can be ill effects on their future children. An example provided was of a person watching over farm animals that suddenly were spooked for unknown reasons, with this shock transmitted and incorporated into that individual. Another example was exemplified during the final genetic counseling session when another child in the room started crying without a clear trigger—this was interpreted as a potential frightening that would stay with her for the rest of her life, unless there was an intervention by a curandero to bring her spirit back to her body. However, neither of the two sets of parents could remember specifically performing an evil deed or being associated with a negative episode in the past, and they were confused as to who and why someone would place a curse on them.

After the deaths of their children, these two sets of parents have acted differently with respect to their traditional medicine beliefs, even though they are closely related to each other and appear to be similarly acculturated. The parents of patients A1 and A3 have continued to access traditional healers, as it was explained to them that reversal of their family’s curse was possible. In contrast, the parents of patient A2 have not continued to seek the advice of healers. They felt that because prayer and rituals did not cure their child, perhaps unexplainable circumstances and “bad luck” were the reasons behind their child’s disease instead. The underlying reason for this difference in behavior is thought to be due to the fact that the parents of patient A2 already have a healthy child.

#### Family B

For the parents of patient B, the disfiguring skin condition first manifested in their older daughter born in Oaxaca. The family was advised by a traditional healer at that time that the mother had a frightening in the past, which was then passed on to her child. Ceremonies that were suggested included prayers and going to a sacred area located on a local mountain. Traditional healers were not utilized to prevent the frightening from affecting their subsequent children, but instead used to help manage pregnancy-related hyperemesis symptoms. The parents had several children born afterwards without any skin diseases, and they had no explanation or reasoning as to why this was the case. After the birth of patient B with her diagnosis of lamellar ichthyosis, the parents were confused as to why only two of their children have this disease and to some extent still believe that the previous frightening event was a contributing factor. However, because of their higher degree of acculturation as well as greater acceptance of Western medicine, they also expressed that bad luck or perhaps the influence of a more omnipotent being could be responsible.

*Lessons learned*: (1) It is important to accept and expect that these families subscribe to a belief system integrating both Western and traditional medicine principles as part of their worldview which incorporates alternative explanations and therapies for their child’s medical condition (Barragan et al. 2011; Barron et al. 2004; Gonzalez-Vazquez et al. 2016; Mittman et al. 1998; Olney and Olney 1993; Penn et al. 2010; Shaw and Hurst 2008; Smoot III et al. 1988; Solomon et al. 2012). (2) The family’s degree of acculturation should be determined early during the interactions, which then calibrates the clinician and counselor’s expectations regarding the extent to which each family places their trust in one medical or belief system versus another (Barragan et al. 2011; Kelly 2009). (3) Accommodations should be made whenever possible to the family’s requests as they pertain to their traditional medicine beliefs, such as inviting healers to lead prayers or perform ceremonies that may require special arrangements that deviate from typical conduct in an inpatient setting (Kelly 2009).

### Discussion of Medical Genetics Concepts

#### Family A

The parents of patient A1 were counseled regarding genetic concepts, autosomal recessive inheritance, and recurrence risks shortly after their child was identified with severe MTHFR deficiency. The Mixteco Bajo interpreter advised us that there was no direct translation of the word “gene,” and thus the English word was used. Attempts were made to verify their level of understanding of the genetic topics that were presented, such as asking them to repeat the information back to us in their own words. These attempts were unsuccessful for reasons noted in the “Social Interactions and Building Rapport” section above. Discussions centered around health education regarding inborn errors of metabolism, and disease states in general proceeded in a similarly unsatisfactory manner. When these same conversations were conducted with the parents of patient A2, the interactions were even less ideal, as they mainly occurred in the PICU setting. Overall, the clinical staff were not confident that any meaningful proportion of the important medical and genetic information was being understood by these parents.

During the final genetic counseling session with the parents of patients A1, A2, and A3, a different approach was chosen. Genetic terms, basic principles of genetics, and Mendelian inheritance patterns were not brought up. Instead, this discussion utilized the framework and concepts matching what the parents already believed. The concept of “bad blood” was expanded upon by stating that “blood” can be “good” (as a surrogate for the wild type alleles) or “bad” (as a surrogate for the pathogenic variants), with the heterozygous carrier state described as the blood being “kind of bad.” It was then discussed that in some instances, the mixing of blood from the parents could indeed lead to disease in a child. We further attempted to incorporate their traditional medicine beliefs by stating that one way of viewing “kind of bad blood” is that it is similar to a curse. However, this curse has not been placed on them from external and supernatural sources. Instead, all individuals carry with them a proportion of kind of bad blood which, if mixed in a particular combination between the parents, results in a child developing a disease. More in-depth details about physiologic processes, inborn errors of metabolism, and neurologic decompensation in MTHFR deficiency were not discussed, but rather folded into the general discussion about the state of their child’s blood. This counseling session was not intended to present complete medical and genetic information. Instead, the primary goal was to try to alleviate the guilt and soul-searching that the parents most likely were and are continuing to experience, perhaps lessening their need to determine exactly how and why they were cursed.

This approach also served as an entry point to counsel them regarding recurrence risks. A pie chart was used, divided into four equal quadrants, and one quarter was filled in to visually represent a 25% recurrence risk of having an affected child if the parents’ blood mixed in the wrong way. It was emphasized that neither set of parents were guaranteed to only have children with this severe disease as illustrated through this pie chart. The parents of patients A1 and A3 were counseled that they had bad luck leading to both of their children being affected, while the parents of patient A2 had less bad luck, supported by the fact that they already have a healthy daughter.

#### Family B

For the parents of patient B, the medical and genetic concepts were presented in a similar manner during the outpatient genetic counseling session after the diagnosis of lamellar ichthyosis was molecularly confirmed. As noted previously, the parents did not have a satisfactory explanation as to why only two of their children had this skin disease. The role of “luck” and different mixtures of the parents’ blood with each of their children, along with the 25% recurrence risk visually represented by the pie chart, served as the main points of discussion with the family. Overall, the parents appeared to understand and seemed receptive to the information presented, likely because of their different degrees of acculturation as well as the fact that they have several other unaffected children.

*Lessons learned*: (1) If it is suspected that there is a low level of health literacy as it relates to Western medicine, attempts should be made to incorporate the family’s traditional medicine beliefs, such as curses and bad blood, into a simplified Western medicine-oriented genetic counseling approach regarding genes, patterns of inheritance, and recurrence risks (Parker et al. 2003; Saleh et al. 2009). (2) There may be different expectations for how much clinical information the medical and genetic staff want to provide, compared to how much the family wants to hear and understand. The case may be that the primary focus of the genetic counseling should be aimed at addressing the parents’ psychosocial well-being, whereby providing too many details will be counter-productive and detract from this goal (Kelly 2009). (3) Differences in level of acculturation, and if the parents have unaffected children, factor into their level of acceptance and understanding of the genetic counseling information presented.

## Strengths and Limitations

The strength of this case study is the fact that a co-author, L. Vasquez Santos, is familiar with several different dialects which matched that spoken by the two families reported herein. He is also the Executive Director of the Fresno-based Binational Center for the Development of the Indigenous Communities and is deeply involved in community advocacy and outreach efforts. Having him as a cultural broker allowed us to more fully explore and understand the Mixteco indigenous community and their beliefs, culture, and worldview. With his planning and input, we could then aim to transmit medical genetics concepts in the best way possible. There are inherent limitations in applying the lessons learned from two families more broadly to an entire indigenous population from Oaxaca. Their thoughts and practices may not necessarily represent common beliefs, and additionally, there will be different levels of acculturation.

## Practice Implications

It is important to recognize early in the clinical interaction that the family is of indigenous background and to verbalize familiarity, knowledge, and acceptance of this information. An interpreter familiar with their dialect, matched at least by distrito (district) and more ideally by municipio (municipality) should be used if possible. Available to the immigrants from Mexico in the USA are assistance and services for additional dialects and cultures under the umbrella of CBDIO (http://centrobinacional.org) (Triqui and Zapoteco, in addition to Mixteco), and many organizations [e.g., Frente Indigena de Organizaciones Binacionales (FIOB) and Mixteco Indigena Community Organizing Project] are available for the Zapoteco and other communities predominantly present in Southern California. Through these organization and others, there is access to a larger pool of interpreters by phone if an in-person interpreter is not available. Questions regarding the utilization of traditional medicine and subscription to non-Western medicine beliefs should be posed early and without any inherent bias. Identification of a cultural broker may be the main barrier to providing optimized genetic counseling services. However, once a cultural broker is recruited and after all members of the clinical team are appropriately trained, it can be determined which of the elements described in this article can be broadly applicable versus individualized to specific cultural practices, beliefs, and worldviews. The family’s degree of acculturation also should be assessed so that the clinical staff can calibrate their expectations regarding how the conversations will proceed. Additionally, it needs to be considered that the psychosocial well-being of the family may be of greater importance than being comprehensive and complete with the information being discussed. The techniques and methods described can be applied to other aboriginal and indigenous populations worldwide.

## Research Recommendations

It would be interesting to explore the genetic counseling outcome if a family’s dialect could only be imperfectly matched, which is a situation likely to occur if there are limited local resources to access or if a multilingual speaker of a more geographically isolated dialect is not immediately available. Patient and family satisfaction scores, as well as the level of understanding of the genetic topics presented, could be assessed after counseling with the best available interpreter at the time. These measurements could then be compared to those obtained after a later session that occurs when the ideal cultural broker is identified and brought into the visits. Another research recommendation is to explore more deeply the worldview of indigenous families who incorporate both traditional and Western medicine. If there are circumstances in which both versus one or the other are used, it would be valuable to delve into the underlying reasons. A better understanding could lead to improved clinical and counseling care. There also could be the expectation of a gradual shift in the utilization of one belief system at the expense of the other because of the pervasiveness of the Western culture within which they now live. Besides subtle shifts that occur over time, medically significant sentinel events could lead to dramatic changes in their worldview.

## Conclusions

The clinical and genetic care of these two families of indigenous background originating from Mexico, combined with education from indigenous advocacy groups and cultural learning centers, have enriched and deepened our understanding of their language diversity and culture and improved our abilities as clinicians in serving this community. These cases illustrate the complex interactions between clinical genetics and region-specific cultural and societal norms and beliefs. Clinical staff need to be aware of, and adapt to, differences in social etiquette, interpersonal interactions, and worldview that prominently features traditional medicine. This adaptation may include abandoning some aspects of Western medicine and genetic counseling training and experience, and engaging the family without the expectation of a robust bidirectional conversation and without many “hard” concepts (genes, Mendelian inheritance, pathophysiologic processes, molecular information, etc.) being discussed. The overall goal should be to “meet in the middle” and normalize the socio-cultural differences between the clinicians and the family, improving the transmission of the clinical and genetic information, thus providing effective and comprehensive genetic counseling and care.
